# 1817. Skin and Soft Tissue Infection Incidence Before and During the COVID-19 Pandemic at A Large Safety Net Healthcare System

**DOI:** 10.1093/ofid/ofad500.1646

**Published:** 2023-11-27

**Authors:** Prudencio Merino, Deborah Kupferwasser, Evelyn A Flores, Donna Phan Tran, Abisay Ortega, Loren G Miller

**Affiliations:** Division of Infectious Diseases, the Lundquist Institute at Harbor-UCLA Medical Center, Torrance, CA, Torrance, California; The Lundquist Institute for Biomedical Innovation at Harbor UCLA Medical Center, Torrance, California; Division of Infectious Diseases, the Lundquist Institute at Harbor-UCLA Medical Center, Torrance, CA, Torrance, California; Division of Infectious Diseases, the Lundquist Institute at Harbor-UCLA Medical Center, Torrance, CA, Torrance, California; The Lundquist Institute for Biomedical Innovation at Harbor UCLA Medical Center, Torrance, California; David Geffen School of Medicine at UCLA, Torrance, California

## Abstract

**Background:**

Skin and Soft Tissue Infections (SSTIs) are one of the most common bacterial infections that drive persons to seek medical care. We hypothesized that during the COVID-19 pandemic, SSTI rates would significantly decrease due to Public Health directives to avoid unneeded care and due to attenuated risk behaviors, such as skin injury and person to person contact, that drive SSTIs.

**Methods:**

We performed a health system wide retrospective study using databases from the Los Angeles Department of Health Services, the second largest U.S. safety net healthcare system which is composed of 4 major medical centers and 20 clinics and ambulatory care centers. We examined all patients who had ≥1 medical encounter (inpatient, Emergency Department [ED], outpatient, or observation) for an SSTI. SSTIs were identified using ICD-10 diagnosis code between March 16, 2017 and March 15, 2022. Pre-pandemic and intra-pandemic SSTI rates were compared using a student’s t-test.

**Results:**

During the study period there was a mean of 426,995 empaneled of patients per month. In total there were 72,218 SSTIs, 36,167 in the outpatient setting, 21,035 in the ED, 1143 in the observation unit, and 13,433 in the inpatient setting. Intra-pandemic SSTI rate was significantly lower than pre-pandemic (2.3 cases/1000 empaneled patient-months versus 3.3 cases/1000 empaneled patient-months, p< 0.0001). SSTI subgroups stratified by site of care all had significant SSTI decreases between the intra-pandemic and pre-pandemic time periods (inpatient (0.5 vs 0.6 empaneled patient-months), observation unit (0.05 vs 0.07), ED (0.6 vs 1.0), and outpatient clinics (1.2 vs 1.6), p< 0.0001 for all comparisons, see Figure).

Skin and Soft Tissue Infection Rates Pre- and Intra- COVID-19 Pandemic

**Figure:**
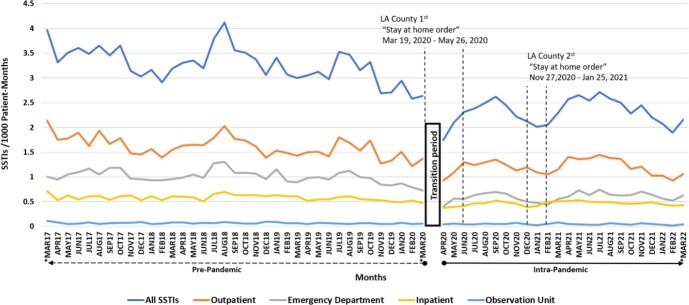

**Conclusion:**

Compared to the pre-COVID-19 pandemic period, SSTI rates in our large U.S. safety net healthcare system significantly decreased by over 30% during the COVID-19 pandemic. Decreases occurred at all levels of care delivery. Whether the results of this study reflect a true reduction in SSTI incidence rates or a reduction in health system utilization for SSTIs requires further examination. Our data provide some insights as to expected changes in common bacterial infections during subsequent pandemics.

**Disclosures:**

**Loren G. Miller, MD MPH**, ContraFect: Grant/Research Support|GSK: Grant/Research Support|Medline: Grant/Research Support|Merck: Grant/Research Support|Paratek: Grant/Research Support

